# How Uncertainty Is Talked About in Language Discordant and Concordant Health Consultations: Patterns of Epistemic Adverb Use and Communication Accommodation Strategies

**DOI:** 10.1111/hex.70873

**Published:** 2026-09-14

**Authors:** Vanda Nissen, Renata F. I. Meuter, Michelle Riedlinger

**Affiliations:** ^1^ Institute for Social Science Research The University of Queensland (UQ) Brisbane Australia; ^2^ Faculty of Health, School of Psychology and Counselling Queensland University of Technology (QUT) Brisbane Australia; ^3^ Faculty of Creative Industries, Education and Social Justice, School of Communication Queensland University of Technology (QUT) Brisbane Australia

**Keywords:** Communication Accommodation Theory (CAT), culturally and linguistically diverse (CALD)‐patients, emergency department, epistemic adverbs, health communication, health practitioner, patient

## Abstract

**Introduction:**

In this study, we explored how uncertainty was expressed through epistemic adverbs in health consultations where patients and health practitioners (HPs) spoke the same language (language‐concordant) versus when they did not (language‐discordant). Further, we applied Communication Accommodation Theory (CAT) to identify, in these settings, how HPs used communication strategies and their patients' communicative responses.

**Method:**

Fifty‐three health consultations between bilingual and monolingual patients and their HPs in a metropolitan emergency department were recorded and transcribed, 39 of which contained epistemic adverbs (e.g., *probably, maybe*). Epistemic adverbs were identified and classified, and the surrounding interactional sequences were analysed using CAT.

**Results:**

Analyses revealed overall greater use of uncertainty adverbs versus certainty adverbs. CAT‐informed analysis identified approximation, discourse management, interpersonal control, emotional expression and interpretability when HPs or patients expressed uncertainty. HPs responded through clarification, reformulation, backchannelling and reassurance. However, in some cases, the interaction did not establish whether the expressed uncertainty had been fully resolved or understood.

**Discussion:**

In the analysed consultations, epistemic adverbs functioned as linguistic markers of uncertainty related to clinical information such as symptoms, timing and possible course of action. Although use of accommodative strategies was evident in all analysed excerpts, in some cases the available data could not confirm whether the bilingual patients had fully understood the information.

**Conclusion:**

Attending to patients' uncertainty as expressed through epistemic adverbs may help HPs accommodate more effectively to their patients' communicative needs.

**Patient or Public Contribution:**

Patients and members of the public were not directly involved in the design, conduct, analysis or reporting of this study. The research was based on the analysis of recordings of real‐life clinical consultations. Because the study focused on evaluating communication patterns within existing consultation recordings rather than developing or testing interventions affecting patient care, additional patient or public involvement was not considered methodologically appropriate.

## 1 Introduction

Epistemic adverbs play important roles in communicating various degrees of certainty and uncertainty, and they may be particularly important in healthcare settings. Imagine a doctor‐patient consultation in a hospital. As the patient, how would you rate your chances of going home if the doctor tells you that *You are **possibly** going home tomorrow* vs. *You are **probably** going home tomorrow*? Here we investigate these kinds of doctor‐patient interactions as they occurred in an emergency department (ED). Specifically, we examined how health practitioners (HPs) and their patients responded to uncertainty through their use of accommodative and non‐accommodative communication strategies, focusing on expressed uncertainty in the interactions. Expressed uncertainty was captured by coding for the use of epistemic adverbs. For the purposes of this study, epistemic adverbs are defined as adverbs that convey a stance and include adverbs such as *certainly, likely, maybe,* etc. [1, 2, 3, 4].

Uncertainty penetrates healthcare. While HPs are aware of uncertainty of diagnosis, treatment options and prognosis, their awareness of how well they communicate that uncertainty to patients may be limited [5]. One of the reasons for that is the nature of uncertainty, which can be defined as a subjective perception of ignorance [6, p. 830]. In its metacognitive conception, the existence of uncertainty is determined by both the absence of understanding and the presence of conscious awareness. When a person gains understanding or loses their awareness of ignorance, they move to a different epistemic state, that is, certainty, ignorance or knowledge [7].

The success of health communication depends partly on how HPs manage to communicate uncertainty to their patients. This is not without its challenges. While some patients value honesty and readily accept diagnostic uncertainty [8], other patients view uncertainty negatively and have difficulty accepting it [9, 10], especially when doctors handle clinical uncertainty paternalistically [11]. A patient‐centred approach to communicating uncertainty is important, even more so in language‐discordant consultations because culturally and linguistically diverse patients often have low health literacy in their second language (L2) [12, 13], experience fears in accessing healthcare [14], and face misunderstandings [15, 16]. These patients may perceive the process of decision‐making and (un)certainty of available treatment options differently due to language barriers and cultural differences [17].^1^

Although verbal expressions of uncertainty naturally occur in more than 70% of clinical visits [18], research into epistemic expressions of uncertainty used in language‐concordant [19] and language‐discordant [19, 20] health communication remains limited. The clinical relevance of epistemic expressions is not restricted to HPs' communication of diagnosis, prognosis or treatment [10, 21]. Patients also communicate uncertainty about symptoms, timing and treatment during clinical encounters. This uncertainty is displayed through a range of linguistic means, including epistemic adverbs [22]. For example, a patient who says that pain began *maybe* 2 weeks earlier expresses their degree of certainty of information (in this case timing) that may be relevant to clinical assessment. The use of epistemic adverbs by patients may therefore identify points at which clarification, checking, reformulation or confirmation is warranted. They should be taken as markers of tentative knowledge or commitment.

The nature of epistemic adverbs, adverbs that convey a personal stance, suggests their importance in health communication: HPs and patients may use epistemic adverbs like *maybe*, *probably* and *possibly* when discussing symptoms, prognosis and treatment options. The interpretation of these adverbs may be particularly important in language‐discordant consultations because their meanings do not always correspond across languages. There are differences in their probability strength and/or pragmatic meaning [4]. Consequently, an expression intended to communicate one degree of likelihood may be interpreted differently (far less certain or perhaps more so) by an interlocutor communicating in their second language (L2). Past experimental research, using epistemic adverbs in the context of medical opinions, has shown that individuals differ in their understanding of these adverbs, depending on their linguistic backgrounds [23] and their age [24]. However, use of epistemic adverbs to communicate uncertainty in naturally occurring clinical consultations remains underexplored.

### 1.1 Communication Accommodation Theory (CAT) and Health Communication

Communication Accommodation Theory (CAT) provides a framework for such research. It has been successfully applied to better understand HP‐patient communication in both language‐discordant and language‐concordant consultations [25, 26, 27, 28, 29]. CAT seeks to explain and predict when, how and why individuals engage in interactional adjustments with others, including inferences drawn, attributions made, and evaluations of and responses to communication received [30, 31]. It recognises that communication is shaped by both immediate and broader socio‐cultural contexts and often involves negotiating social category memberships through accommodation. Interlocutors have expectations based on social norms and stereotypes and use specific communication strategies to express their attitudes towards each other and their social groups [32].

Based on the intergroup or interpersonal dynamics that occur in a conversation Gallois et al. [33]) describes five types of communication strategies within the CAT framework, namely *interpretability* (focusing on speakers' conversational competence), *approximation* (adjustment of speech patterns), *discourse management* (management of different environments and contexts), *emotional expression* (empathy and reassurance demonstrated by speakers) and *interpersonal control* (use of power and roles by speakers) [26, 34]. When used effectively, these strategies facilitate communication and also reduce communication barriers [35].

However, communication is not always accommodating. Gallois et al. [33] note that speakers may underaccommodate by maintaining their behaviour and discourse without addressing the needs of other interlocutors or overaccommodate by holding their own stereotypes about interlocutors' groups. A review of studies on HP‐patient interactions found that both parties have problems approximating each other [36]. Patients emphasise that doctors need to listen to them and ask questions to ensure understanding. When they feel that they can communicate, it influences the likelihood that they will [37]. This aligns with experiences of HPs who note that interactions with patients are successful when they manage to listen to and understand them. Non‐accommodative communicative strategies (being rude, dismissive, etc.) are associated with negative experiences for patients [37], as well as for HPs in their interactions with patients [35] and in intergroup interactions with other HPs [38, 39, 40].

Uncertainty can place additional constraints on accommodation in health consultations because interlocutors must negotiate incomplete or probabilistic information while also maintaining clarity and trust. For HPs, this may involve balancing transparency and reassurance with the need to provide an actionable plan; for patients, uncertainty may affect how they interpret information, seek clarification and participate in decision‐making [10, 41, 42]. These demands may make accommodation more difficult, particularly in language‐discordant consultations, where patients communicating in another language may have fewer interactional resources with which to question or contest clinical recommendations [43].

In the present study, epistemic adverbs were treated as linguistic markers of uncertainty and likelihood. Rather than examining the adverbs in isolation, we applied CAT to the surrounding interactional sequences to explore how HPs and patients responded through accommodative and non‐accommodative practices. Specifically, we analysed naturally occurring language‐concordant and language‐discordant consultations recorded in an Australian metropolitan ED to examine how uncertainty expressed through epistemic adverbs was managed in interaction. To our knowledge, this is the first study to apply CAT specifically to such interactional sequences.

## 2 Methods

### 2.1 Participants

The participants consisted of HPs and patients, recruited in the ED of a Metropolitan hospital in Brisbane. Each group was comprised of monolingual and bilingual/multilingual speakers, as follows.

*Health practitioners*. The HPs (*n* = 37) consisted of doctors, nurses and physiotherapists working at the ED. Two‐thirds of the HPs were monolingual English speakers (*n* = 25). Most of the doctors were registrars.

*Patients*. All patients (*n* = 43) were recruited in the Ambulatory Care area of the ED. Only patients who were identified in triage as meeting inclusion criteria (i.e., *National Triage Scale* 4 or 5) were considered. The National Triage Scale (NTS) for Australian Emergency Departments is used by most EDs to identify patients care priorities. Every patient is assigned a triage category based on their medical problem and the need for healthcare. The NTS is a five‐point scale. Categories 4 and 5 are classified as semi‐urgent and non‐urgent, respectively [44]. We worked with the duty nurse to establish which patients would be seen by participating HPs. We approached those patients in the waiting area to explain the research and establish if they wished to participate. Upon obtaining the patient's consent, we alerted the respective HP.

## 3 Materials

### 3.1 Language Background Questionnaire (LBQ)

The LBQ captured basic information about participants (e.g., age, gender, country of birth, country of residence), as well as details of their language background (e.g., self‐reported proficiency in speaking, listening, reading and writing in each language), employment status and highest level of educational attainment.

### 3.2 Procedure and Data Collection

HPs and patients were informed about the study, and all provided consent prior to their participation. HPs learned about the research via email, notices and through daily scrums (brief staff meetings used to discuss priorities, workload and operational issues). All participants completed the LBQ. The HPs completed the LBQ on the day of recording (only once if they were recorded in more than one consultation). Patients completed the LBQ before or after their consultation. All patients also completed the post‐consultation questionnaire.

*Data Collection.* Real‐life consultations between patients and their HPs were video‐ and audio‐recorded. Most consultations occurred in private areas of the ED. All participants agreed to both audio‐ and video‐recording, and reassurance of de‐identification was provided. Before the start of the consultation, the researcher set up the camera, started the recording and then left the room. HPs were asked to make sure that privacy was maintained. Physical examinations were not recorded. The data were collected across a 2‐month period in 2021. Overall, 53 consultations involving monolingual and bilingual HPs and patients were recorded.

### 3.3 Data Analysis

The recordings were transcribed verbatim by a professional transcriber and then checked by the research team. An uncertainty episode was defined as an interactional sequence that contained an epistemic adverb with the immediately surrounding utterances that contextualised the focus of the uncertainty. The transcripts were coded twice by the same researcher (V.N.), once for the occurrence of epistemic adverbs and once for communication strategies used by the HPs. A CAT strategy was coded as linked to the epistemic adverb when the subsequent response addressed the proposition or degree of certainty marked by the adverb, for example through clarification, repetition, reformulation, reassurance, correction or topic redirection. More than one CAT strategy could be coded within an episode.

The resulting coding was then checked by the third author (R.M.), and any discrepancies in coding were resolved by agreement between these two authors. For the purposes of this study, we excluded interjections (e.g., ‘um’), unclear words, and words that did not form part of the direct communication between the patient and their HP but were intended for others (e.g., other HPs or companions). To understand how patients and HPs talked about and negotiated their understanding of uncertainty and risk, first we identified and linguistically classified occurrences of epistemic adverbs. Second, we coded those parts of the consultations that contained epistemic adverbs and explored the CAT‐strategies [33, 45] used by HPs and patients as linked to the use of (un)certainty adverbs. The coding system for CAT strategies is provided in Table 1.

**Table 1:** CAT strategies with examples of accommodative and non‐accommodative use.

Strategy	Type of accommodation	Examples
Approximation	Accommodation	Indicating that they follow the conversation by adjusting their talk appropriately
	Non‐accommodation	Not adjusting their talk or over‐adjusting (e.g., by using exaggeratedly slow speech)
Discourse management	Accommodation	Managing conversation by maintaining topic and backchanneling, listening, inviting questions from communication partner
	Non‐accommodation	Dominating conversation, not listening, not letting others direct conversation
Interpersonal control	Accommodation	Treating each other as equals, sharing control over conversation, maintaining appropriate power dynamics
	Non‐accommodation	Being authoritative or too personal, treating other like a subordinate
Emotional expression	Accommodation	Attending to emotional needs of communicative partner, showing empathy, providing reassurance
	Non‐accommodation	Not showing empathy or too much empathy, using humour inappropriately, not recognising the other's emotional needs
Interpretability	Accommodation	Explaining things clearly and concisely, checking understanding
	Non‐accommodation	Using complex terminology, ignoring communication partner's conversational competence, not adjusting to cognitive and psychological needs of communication partner

## 4 Results

For the purpose of our analyses, we excluded transcripts where no epistemic adverbs were identified (*n* = 11),^2^ consultations that were brokered (i.e., when patients had limited English and conversations mainly occurred between companion and HP [*n* = 2]), and one consultation that mainly concerned financial matters. In three consultations, a student nurse also attended but only observed; these consultations were retained. In all, 39 consultations were retained for analysis. The consultations encompassed bilingual and monolingual HPs and patients. This enabled the analysis of conversations between (1) monolingual speakers of English (14 consultations; language concordant); (2) bilingual speakers of English as their second/other language (3 consultations; language discordant); and (3) monolingual and bilingual speakers of English (22 consultations; language discordant) (see Table 2 for an overview). In 10 consultations, patients were accompanied by a family member, friend or chaperone.^3^

**Table 2:** Overview of the 39 conversations retained for analysis, presented according to the speakers' language background (bilingual or monolingual). The number of participants (*n*) in each group is presented in brackets.

	Patients		
	Bilingual (*n* = 11)	Monolingual (*n* = 23)	Total
HPs			
Bilingual (*n* = 10)	3	12	15
Monolingual (*n* = 18)	10	14	24
Total consultations	13	26	39

The 39 conversations retained for analysis ranged between 1.52 min and 23.48 min in length (total length ≈ 368 min; mean length = 9.43 min). The consultations were spontaneous, real‐life ED interactions and were not guided by a study‐specific consultation protocol. Consultation topics and lengths varied according to patients' presenting concerns, HPs' roles, and the clinical circumstances of the ED, including COVID‐19‐related restrictions. There were 28 HPs, each of whom engaged in one to five unique consultations, and 11 of whom were bilingual speakers of English. Of the 34 patients who took part, one third (*n* = 11) spoke English as their second/other language (L2) with different levels of proficiency (see Table 3). All but two monolingual English‐speaking patients were born in Australia, with New Zealand and Scotland being the other birthplaces. The bilingual patients were all born overseas, and spoke Afrikaans (*n* = 1), Chinese^4^ (*n* = 1), Greek (*n* = 1), Italian (*n* = 2), Korean (*n* = 2), Polish (*n* = 1), Samoan (*n* = 1), Shona (*n* = 1) and Vietnamese (*n* = 1).

**Table 3:** Patient and HP demographics, including mean self‐rated English proficiency in speaking (S), reading (R), writing (W), and listening (L) (1 = no ability at all; 5 = native‐like ability), and gender (female [F] and male [M], with mean age in brackets).

	English proficiency				Mean overall proficiency	Gender	
	S	R	W	L		F	M
Patients							
Bilingual (*n* = 11)	3.9	4.2	3.8	3.8	3.9	7 (41)	4 (65.2)
Monolingual (*n* = 23)	5.0	5.0	5.0	5.0	5.0	7 (54)	16 (52.0)
HPs							
Bilingual (*n* = 11)	4.8	4.7	4.5	4.9	4.7	2 (31)	9 (29.0)
Monolingual (*n* = 17)	5.0	5.0	5.0	5.0	5.0	12 (38)	6 (34.0)

The total number of epistemic adverbs across 39 conversations was 164. This count excludes any immediate repetitions.

### 4.1 Patient Talk

The cohort consisted of 34 patients (age range = [20, 80], M = 51.82, SD = 18.92) (female, *n* = 14). An overview of the patients' talks is provided in the Supporting Information S1: Appendix. During the talk with their HP, in total our patients used 94 epistemic adverbs to reflect their degree of certainty. The epistemic adverbs were used to describe current symptoms and medical history (*n* = 56), and to answer time‐related questions (*n* = 30) and lifestyle‐related questions (*n* = 8).

Uncertainty adverbs (total = 91) were the most used adverbs (97% of the total number of adverbs). Amongst the uncertainty adverbs, *probably* was the most prevalent adverb (total = 59; 57%), followed by *maybe* (total = 37; 36%). The uncertainty adverbs *likely* and *possibly* were used sparingly (three times and once, respectively) (see Table 4).

**Table 4:** Number of epistemic adverbs used, provided separately for the patients and the HPs, by language background (bilingual vs. monolingual). Certainly (C), definitely (D), obviously (OB), of course (OC), likely (L), maybe (M), perhaps (PE), possibly (PO), probably (PR).

	Certainty adverbs (*n* = 3)				Uncertainty adverbs (*n* = 91)					
	C	D	O	OC	L	M	PE	PO	PR	Total (*n* = 94)
Patients										
Bilingual (*n* = 11)	—		—	1	—	6	—	1	11	19
Monolingual (*n* = 23)	1	1	—	—	3	23	—	—	47	75
Total	1	1	—	1	3	29	—	1	58	94

	Certainty adverbs (*n* = 20)				Uncertainty adverbs (*n* = 50)					
	C	D	O	OC	L	M	PE	PO	PR	Total (*n* = 70)
HPs										
Bilingual (*n* = 11)	—	4	1	—	—	7	—	—	9	21
Monolingual (*n* = 17)	2	2	5	6	1	5	1	—	27	49
Total	2	6	6	6	1	12	1	—	36	70

*Note:* Patients: Mean occurrence/conversation for *maybe* (bilinguals = 1; monolinguals = 0.88); mean occurrence/conversation for *probably* (bilinguals = 0.86; monolinguals = 1.8). HPs: Mean occurrence/conversation for *probably* (bilinguals = 0.82, monolinguals = 1.55); mean occurrence/conversation *maybe* (bilinguals = 0.64, monolinguals = 0.28); mean occurrence/conversation *definitely* (bilinguals = 0.36, monolinguals = 0.11).

### 4.2 Health Practitioner Talk

The cohort consisted of 28 HPs (age range = [22, 62], M = 34.14, SD = 11.56) (female, *n* = 14), and included doctors (*n* = 19), nurses (*n* = 7) and physiotherapists (*n* = 2). As was the case for our patients, the adverbs *probably* and *maybe* were also the most used adverbs for the HPs (36 and 12 occurrences, respectively; see Table 4). Overall, HPs used more certainty adverbs (*n* = 20) than patients (*n* = 3). The adverbs *of course* (*n* = 6) and *certainly* (*n* = 2) were only used by monolingual HPs (see Table 4), who were also the only ones to use *likely* and *perhaps*. Interestingly, not all HPs expressed their uncertainty through epistemic adverbs, with five monolingual and six bilingual HPs not using any epistemic adverbs. They talked about uncertainty using other linguistic means, such as modal verbs (e.g., *can, may, could*); hedges (such as *sort of*), and epistemic parentheticals (e.g., *I think*).

### 4.3 Communication Accommodation Strategies

Our exploration of CAT strategies focused on their use only during those sequences in the consultations when epistemic adverbs were produced to signal a degree of (un)certainty by the patient and/or the HP, or where it was evident that something was unknown or unclear. CAT strategies were evident in all 39 conversations and, as it transpired, their use was largely targeted either at addressing uncertainty expressed by patients or at managing the HPs' expressed uncertainty about their clinical knowledge. Overall, HPs applied the full range of CAT strategies to accompany their use of epistemic adverbs: approximation, discourse management, interpersonal control, emotional expression and interpretability. We will identify these strategies as they occurred in consultations and discuss the role of epistemic adverbs in accommodation/non‐accommodation.

*Approximation.* When discussing risk and uncertainty, HPs demonstrated accommodative behaviours by listening to and watching their patient's verbal (‘mmm’, ‘yeah’) and nonverbal expressions and indicating that they were hearing/following by adjusting their talk accordingly (approximation). To enhance mutual understanding, sometimes HPs also adopted common words used by their patients. Table 5 is an example of a language‐discordant consultation where a bilingual doctor picked up on his patient's uncertainty and matched her vocabulary.

**Table 5:** Interaction between a monolingual patient (female; age 32) and a Korean‐English bilingual doctor (male; age 25).

Patient:	Yeah, I've got a headache, yeah. Um, so I guess, um, like I, I felt like I was constipated towards the start of the week. But um, I've still been able to go regularly, but it's been like harder to push um, and like, I, like it feels like I'm blocked but I've still been able to go.Mm‐hm.
Doctor:	Yeah.
Patient:	So would you say like it's more bloating, or?
Doctor:	No, it's not bloating.
Patient:	Ok.
Doctor	Nup.
Patient:	Ok. Feels constipated. So, like as in stool's more dry, and harder?
Doctor:	Harder to push, yeah.Push.
Patient:	Um, even though I've still been able to go regularly. So like I don't think I'm constipated, but it feels like I am.
Doctor:	Oh ok. Yeah.
Patient:	That's *probably* a the only way I can describe that, yeah.
Doctor:	Yeah. But it's not like you're completely blocked up. (JK_DO_KM_24062021)

^a^ Italics are used to highlight the use of epistemic adverbs.

In Table 5, the patient used ‘constipated’ and ‘blocked’ interchangeably. Further in the conversation, she again used the word ‘constipated’ to describe her condition but did not believe that was what she had. Her use of *probably* along with other epistemic means, such as approximators *like* (‘it feels like I am’) and *I don't think*, indicated her uncertainty regarding the nature of her symptoms. The bilingual doctor responded to this uncertainty by backchanneling (e.g., ‘yeah’, ‘Ok’ etc.) and by adopting the patient's terminology, that is, also using the descriptors ‘constipated’ and ‘blocked’, whilst providing a possible description of those terms. The patient responded by providing further explanations. The doctor's use of the colloquial word ‘blocked’ illustrates approximation, which enhances mutual understanding by aligning the HP's language with that of their patient. Similar strategies have been reported in Jain and Krieger [46] study, which found that international medical graduates' use of approximation strategies to overcome, verbally, the more subtle language barriers includes building a colloquial vocabulary of slang words, idioms and other popular words. This strategy is successfully demonstrated in the above example.

*Discourse Management*. Discourse management includes different ways of managing a conversation by, for example, taking the lead or sharing topic selection, (not) allowing turn‐taking, etc. [47]. In the recorded conversations, HPs maintained conversations with their patients by using, for example, backchanneling and topic maintenance. In Table 6, a bilingual doctor demonstrated understanding of his patient's problem and managed discourse by maintaining the topic. He made a note of her symptoms (dizziness and nausea) and reminded her of these when she seemed more concerned about another, medically less concerning, symptom (pain in her calves).

**Table 6:** Interaction between patient (female; age 51; monolingual English) and doctor (male; age 26; bilingual Korean‐English).

Doctor:	Any um, any nausea or vomiting?
Patient:	Dizziness and feeling a bit nauseous. No vomiting.
Doctor:	Ok.
Patient:	No.
Doctor:	Yeah. Any changes to how much you've been eating?
Patient:	No.
Doctor:	No. And any colds, or sore throats, colds?
Patient:	No.
Doctor:	No.
Patient:	No.
Doctor:	Um, and no urinary symptoms.
Patient:	No.
Doctor:	So not needing to go more often than usual, and not burning.
Patient:	Not that I'm aware of. Yeah.
Doctor:	Any swelling or pain in your calves?
Patient:	No, I've been checking that.
Doctor:	Yeah.
Patient:	It's a bit sore, but I have done a fair bit of walking this week, which normally niggles it.
Doctor:	Yep
Patient:	Um, but yeah, I've been fairly, watching things closely. This was *probably* the most significant thing.
Doctor:	Yeah, and you've been lightheaded and dizzy.
Patient:	Yeah. (JB_DO_LL_18062021)

In Table 6 above, both speakers used backchanneling (e.g., ‘yeah’). The doctor noted his patient's hesitance when she was evaluating the nature of her symptoms (‘This [soreness in calves] was *probably* the most significant thing’). He responded by refocusing the discussion on other symptoms she had reported (lightheadedness and dizziness) that could reflect more serious health issues, showing good use of discourse management. Table 6 also shows the HP's use of interpretability. The doctor successfully sought information from the patient, who demonstrated understanding by answering the doctor's questions.

We observed several types of questions used by HPs with older patients, possibly to elicit further or more nuanced information from the patients and support mutual understanding. Table 7 illustrates how a patient's use of epistemic adverbs can mark uncertainty concerning symptom description, and how an HP can respond by eliciting further information and reformulating the patient's account. The patient used two epistemic adverbs: first, he used *probably* when trying to identify the source of his pain. The doctor responded with ‘Yeah?’, inviting the patient to elaborate, and subsequently offered a provisional formulation of the pain location: ‘You felt the pain within your hip?’. The patient then indicated uncertainty about the cause of the pain by saying that he ‘didn't know whether it was muscular, or what’. When asked about symptom onset, he used *maybe* to qualify his estimate of ‘3 or 4 weeks ago’. His uncertainty was picked up by the HP, who then asked: ‘3 or 4 weeks ago you started having pain? In the hip?’. Rather than replacing the patient's tentative estimate with a more definite account, the HP's responses preserved the uncertainty conveyed by *maybe* while making the timing and location available for confirmation or correction. This sequence combined discourse management, through the elicitation and maintenance of the symptom account, with interpretability, through repetition and reformulation.

**Table 7:** Interaction between patient (male; age 73; monolingual English) and doctor (male; age 32; bilingual Malay‐English).

Patient:	Well, *probably* from, I've always had a bit of a dodgy back.
Doctor:	Yeah?
Patient:	Yeah, it's just starting to get in, in my felt, I felt it is in my hip, right.
Doctor:	You felt the pain within your hip?
Patient:	Yeah, I felt that it was just here in my hip.
Doctor:	Yup.
Patient:	And I didn't know whether it was muscular, or what. I went and had ah, you know, a couple of massages. Um, you know, deep tissue massages.
Doctor:	Yep. When did that start? When did the pain start?
Patient:	Hmm, maybe 3 or 4 weeks ago
Doctor:	3 or 4 weeks ago you started having pain? In the hip?
Patient:	Mmm. And then uh, it got worse and worse, and um, I couldn't, I couldn't lay on my back. I couldn't lie on my left side, of course. (JV_DO_GE_21062021)

HPs asked follow‐up questions and checked understanding with their patients. They used several strategies, such as asking patients questions and inviting questions from them, backchanneling, and maintaining the topic. Although time constraints are common in EDs, the HPs in the recorded consultations attempted to answer their patients' questions.

*Interpersonal Control*. In the recorded consultations, the HPs used interpersonal control skills to invite their patients to participate in the consultation, and allowed some sharing and decision‐making control. For example, in Table 8, a monolingual nurse consulted with a bilingual patient who was a nurse herself and also a colleague. She had been attacked by a patient who had twisted her arm.

**Table 8:** Interaction between patient (female; age 37; bilingual Shona‐English) and nurse (female; age 62; monolingual English [UK]).

Nurse:	And you've got to go back to work, or no?
Patient:	No.
Nurse:	You *probably* won't be able to.
Patient:	No. [laughs]
Nurse:	But, you're *probably* driving.
Patient:	Yeah.
Nurse:	Yes.
Patient:	Yeah, I have to drive;
Nurse:	Yes, ok. So would you be happy to settle for something simple, like paracetamol and Ibuprofen?
Patient:	Yeah.
Nurse:	And see how you go? and then if that doesn't help it, then we can get you something a bit stronger.
Patient:	Yeah.
Nurse:	We might have to ring someone to pick you up.
Patient:	Ok. Sure. Thank you. (KR_NU_WC_11062021)

In Table 8, the nurse used *probably* for assessments of the patient's immediate plans, and doing so may have been interpreted by the patient as an invitation to confirm or correct the HP's assessments. Following the patient's statement that she would not return to work, the nurse says, ‘You probably won't be able to’. The patient responded with ‘No’ and laughter, demonstrating agreement with the nurse's assessment. The nurse then formulated a second tentative inference: ‘But, you're probably driving’. In this instance, *probably* made the transport arrangement open to confirmation or correction. The patient confirmed and elaborated her reply: ‘Yeah, I have to drive’.

The patient's response became relevant to the subsequent discussion of treatment and transport. The nurse asked whether the patient would be ‘happy to settle for something simple, like paracetamol and Ibuprofen’, thereby presenting the initial medication plan as an option and inviting the patient's agreement. She then explained that stronger medication could be considered if the initial approach was insufficient and raises the possibility that someone might need to collect the patient. The sequence therefore moved from tentative assessments of the patient's immediate plans to a negotiated plan concerning medication and safe transport. This interaction primarily illustrated interpersonal control and discourse management. The nurse retained responsibility for advising on treatment and safety while using the epistemic adverb *probably* to provide the patient with opportunities to confirm information and express a preference. The patient's confirmation of the driving arrangement is embedded into the subsequent plan of actions. The accommodation is therefore visible not simply in the nurse's use of *probably*, but also in the sequence through which the tentative formulation invites a response and the response informs the developing plan.

Although HPs appeared to encourage their patients to participate in decisions about their health, sometimes the result of these attempts made them appear as if they lacked confidence in giving their professional opinion. For example, in Table 9, after checking her patient's movements, the physiotherapist advises her that she can go home if she is confident to do so.

**Table 9:** Interaction between patient (female; age 61; monolingual English) and physiotherapist (female; age 25; monolingual English).

Physiotherapist:	Yeah. If your symptoms get worse, like getting more and more numbness.
Patient:	Yeah.
Physiotherapist:	You'll just see a doctor.
Patient:	Ok.
Physiotherapist:	Yeah. Do you have any questions for me?
Patient:	No, not what I can think of.
Physiotherapist:	I think, from the mobility perspective, *probably* you can go home, if you are confident for now.
Patient:	Yeah. Well, I spend most of the time in bed anyway.
Physiotherapist:	All right. Thank you. (SS_PT_SS_24052021)

In Table 9, the HP expressed her professional opinion (‘from the mobility perspective’). Her use of *probably*, together with the additional epistemic expression *I think* and the conjunction *if*, might have been an attempt to acknowledge the patient's autonomy in making her own decision about going home, but the patient could equally have perceived it as a lack of confidence. However, the patient agreed with the plan (‘Yeah’).

In the recorded consultations, HPs generally appeared to maintain collaborative interactions with patients. They allowed patients to interrupt them, without experiencing any difficulties with regaining control over the conversation. On one occasion, we noted some power imbalance (Table 9), when a young physiotherapist used several epistemic expressions when providing her professional opinion, which could have been perceived as a lack of confidence by her older patient. However, her use of epistemic expressions is consistent with the findings of a study on physiotherapist‐patient communication showing that, despite guidelines which require clear communication with patients, physiotherapists often used indirect and ambiguous communication to offset the potential negative connotations which can arise when one person needs to comment on physical shortcomings of another person [48].

*Emotional Expression*. Emotional expression strategies attend to the emotional needs of a communication partner. Across our recorded conversations, the HPs were observed to use epistemic adverbs also to validate patients' concerns, provide reassurance and show empathy by using humour to bring some levity to serious and concerning health conversations and situations. Humour can make medical procedures less stressful for patients, as shown in Table 10. Here, the female doctor was struggling to insert a needle into a vein that was difficult to locate.

**Table 10:** Interaction between patient (male; age 45; monolingual English) and doctor (female; age 30; monolingual English).

Doctor:	Tell me it that's too tight or anything.
Patient:	No, that's alright, I'm alright.
Doctor:	I think you've got one here.
Patient:	Yep. No, you can, that one works out sometimes, yeah.
Doctor:	You've got one here as well, which is good. I mean, I say that now, and I'll *probably* jinx myself.
Patient:	Yeah, no, once you pull the needle out it'll run deep and hide.
Doctor:	Yeah. [laughs] (EH_DO_DC_07062021)

In Table 10 above, it is clear that by acknowledging that he has difficult veins (…‘that one works out sometimes, yeah’), the patient makes things less stressful for the doctor. In turn, the doctor used *probably* coupled with the verb ‘jinx’ to make light of the tricky process of finding a vein (which is not a pleasant experience for a patient). The patient's joke in response to her comment shows that he understood and accepted that the procedure was not easy in his case. Both speakers used humour to lighten the situation.

*Interpretability.* Interpretability refers to the ability to explain things clearly and concisely and to check the understanding of the other party. Generally, in our recorded conversations, both interlocutors showed that they considered the other person's knowledge and adjusted their speech accordingly. In the main, epistemic adverbs were used appropriately and enhanced clarity. This was evident in a consultation with an older bilingual patient who had difficulty remembering the term CT when discussing possible procedures for his leg. In Table 11, he used an epistemic adverb, *maybe* along with a word‐search formulation, ‘what is it, what do they call this’ and an uncertainty gesture to display his uncertainty, which helped the nurse to identify the likely procedure he was referring to, which was a CT scan. From a CAT perspective, the extract illustrates interpretability, because the nurse draws on the patient's verbal and non‐verbal cues to provide a possible term, as well as approximation, because she adjusts her response to the patient's communicative difficulty.

**Table 11:** Interaction between patient (male; age 72; bilingual Italian/English) and nurse (female; age 33; monolingual English).

Nurse:	It's easy, isn't it? I don't mind at all. Well, I'll [unclear] the room, and the doctor will see you for your leg, and hopefully he can sort it out for you today
Patient:	Well, *maybe* he does, what is it, what do they call this [gestures]?
Nurse:	CT potentially, yeah. Yeah, we'll see what they say. Yep, all yours. (MG_NU_VI_09062021)

Some HPs used simpler phrasing when speaking with patients with limited English proficiency. This may have supported the clarity of their communication with patients. In Table 12, for example, a nurse started to use simpler words when speaking with an older bilingual patient with limited English proficiency.

**Table 12:** Interaction between patient (male; age 68; bilingual Vietnamese‐English) and nurse (female; age 33; monolingual English).

Patient:	Ahhh, two day, today. Two day, one, two day. I would do *maybe*[?]… 10 o'clock, at night, I think, I [get it on the eye]
Nurse:	Ok, so two days ago.
Patient:	And I woke up, no [unclear], ‘Ohhh.'.
Nurse:	Ok, so 2 days ago, 10 o'clock, shampoo in the eye?
Patient:	Yeah.
Nurse:	And your eye's been sore for 2 days?
Patient:	Yeah, ohh. [gestures with hands in front of face]
Nurse:	Both of them or just one?
Patient:	Two.
Nurse:	Two eyes. Ok.
Patient:	Oh, and [long time it can be]?, and put water[?] in it. [makes eye dropper gesture]
Nurse:	Yup.
Patient:	That sort of colour. [gestures at something across the room]
Nurse:	Oh yes.
Patient:	Those colours.
Nurse:	Yes. So drops, eye drops. Ok
Patient:	Yes. I called my, my… eh… you know they have, with the [unclear]
Nurse:	Ambulance?
Patient:	He go ‘What are you?’ I don't know
Nurse:	It didn't help.
Patient:	Ohh, eye was sore.
Nurse:	Did it help your eye, or no?
Patient:	No.
Nurse:	No. Ok
Patient:	Oh, [crosstalk]
Nurse:	Um, ok, but how's your vision? *Can you see out of your eye?*
Patient:	Yeah.
Nurse:	You *can* see?
Patient:	[nods] (MG_NU_VI_09062021)

Table 12 shows that the (older) bilingual patient used several epistemic expressions, including the adverb *maybe*, when trying to explain what happened to his eye. His use of minimal responses, epistemic expressions and the observed use of gestures (instead of words) showed that the patient had limited English proficiency. The nurse modified her talk and started using simpler phrasing (approximation). For example, she reformulated the question ‘How is your vision?’ as ‘Can you see out of your eye?’. After receiving an affirmative reply, she then checked again using the simpler question: ‘You can see?’. By progressively reducing uncertainty, the HP helped to improve interpretability for this patient. However, because of his limited English, it is not possible to say whether the patient fully understood what he was asked, and the assistance of an interpreter would have been beneficial. The type of communication shown in Table 12 can be an indication for HPs that these patients may not be able to participate fully in a decision‐making process and that they should assure themselves of the patient's understanding before proceeding, as this HP tried to do. Doing so is critically important because a patient's reduced or compromised understanding when exacerbated by a language barrier may result in a treatment that would not be the patient's preferred one had they understood, for example, its implications for their health compared to other available treatment pathways. Consistent with this caution are Hong Kong‐based findings that bilingual patients speaking in their weaker L2 tended to go along with the suggested course of action, while patients using their L1 were more likely to debate and discuss their doctor's recommendations before making their decision [43].

Perceived language‐related differences can also constitute potential barriers for bilingual HPs. While bilingual HPs mostly successfully managed interpretability when talking to their patients, on some occasions it was not always clear if the patient completely understood them. The HPs' word choice and accent, at times, may have hindered full understanding by their patients.

In Table 13, the bilingual nurse explained to his monolingual patient what was going to happen next. Although the patient appeared to understand the main message (i.e., he would receive some Endone for his pain and be seen by a doctor), it was not clear whether he understood the information completely. He only replied: ‘Ok’ and did not ask any questions. Admittedly, the patient was in considerable pain. The nurse's use of the epistemic adverb *probably* was justified on this occasion because he was referring to possible actions of others (doctors). However, his sentence structure and some pronunciation issues (enhanced by the wearing of a mask) may have hindered the patient's ability to fully understand the nurse.

**Table 13:** Interaction between patient (male; age 43; monolingual English) and nurse (male; age 46; bilingual Malayalam‐English).

Nurse:	What number you call for your pain right now?
Patient:	Oh, about 9.
Nurse	Mmm, ok. I've got [unclear] Endone um, the dose of Endone now
Patient:	Mmm…
Nurse:	For the, taking care of just the pain, so I'm going not going to set you're a problem.
Patient:	Hmm.
Nurse:	The doctor will see you and see what they can do, *probably* they will start with the [unclear] of the pain, and depends how it is, and see from there, about how we take care of just the pain with some Endone tablets now.
Patient:	OK [groans]
Nurse:	Ok. Ok. So that is 5 mg of Endone.
Patient:	Yeah. (ST_NU_BS_06072021)

Overall, HPs and their patients tried to accommodate each other in both the language‐discordant and the language‐concordant consultations. When talking to bilingual patients, overall HPs used different communication strategies and accommodated their patients, with just a few instances of over‐ or under‐accommodation. The use of interpreters for consultations with patients with low English proficiency would have enhanced mutual understanding. Bilingual HPs were mostly understood by their patients but, in some instances, sentence structure and/or pronunciations were not clear, and it was not clear whether their patient completely understood the message (see Table 13).

## 5 Discussion

While uncertainty in healthcare has been extensively studied [42, 43, 49], this is the first attempt to examine how uncertainty is negotiated interactionally through the prism of CAT, and, specifically, at points of uncertainty in a consultation (as identified through the use of epistemic adverbs). We examined which epistemic adverbs were used by bilingual and monolingual HPs and their patients in an ED context and how both interlocutors (HPs and patients) accommodated each other when communicating uncertainty using epistemic adverbs. There were several findings, and these are discussed below.

First, both patients and HPs used more adverbs of uncertainty than adverbs of certainty, with *probably* and *maybe* the most used adverbs. This is consistent with Lian et al. [41] observations on the negotiation of uncertainty in clinical encounters. In their qualitative study of 20 medical consultations, patients conceptualised uncertainty mainly indirectly, often with words like ‘think’, ‘maybe’, ‘probably’ and ‘hopefully’. The finding is also consistent with Wierzbicka's [4] claim that *probably* is the most frequent and important epistemic adverb in English. The popularity of *maybe* (in preference to *perhaps*, which has the same meaning) may be explained by the register, with research indicating that *maybe* is more common for spoken English [1].

As previous research shows, even native English speakers from different speech communities (e.g., Canada and Australia) may have a slightly different understanding of words of risk and certainty such as *probably* and *possibly* [23]. There are also indications that epistemic adverbs may be interpreted differently across monolingual and bilingual speakers. For example, bilingual speakers may experience difficulties distinguishing between *maybe*, *perhaps* and *possibly*. Although they are considered synonyms, they convey different levels of formality and possibility [50]. This could be another explanation for why both patients and HPs used *maybe* as a preferred adverb when conveying uncertainty.

Second, among adverbs of certainty, *obviously* and *of course* were the most common ones. This is consistent with Tagliamonte and Smith [51] findings that *obviously* and *of course* are the most common epistemic adverbs across all spheres of spoken English in Canada and the United Kingdom, while *clearly* and *evidently* are very infrequent. They further noted that the use of *of course* declines and that of *obviously* increases, particularly in speech of speakers born after 1980. Although Tagliamonte and Smith [51] did not look at the use of these epistemic adverbs in Australian health communication, we note that—unlike their observations—the adverbs *clearly* and *evidently* were not used by our patients and HPs. At the same time, the adverb *of course* was quite common among monolingual HPs (*n* = 10), suggesting a higher level of confidence in HPs compared to their patients.

Third, although the communication we observed was mostly accommodative, there were instances where the patient's understanding was in doubt (see Table 13). Whilst these examples of possible (if slight) misunderstandings may not have had a significant impact on the patient, it is important to note that clarity of health communication is important, especially when one of the parties is bilingual. A US study found that culturally and linguistically diverse (CALD) patients often seek linguistic accommodation from HPs, while doctors similarly expect patients to adjust their talk to be clear and understandable [52]. This was supported by the findings of a recent study on patients' preferences for their doctors' communication strategies across cultures, which found that interpretability was the most frequently mentioned communication strategy for all countries, suggesting that all participants valued being able to understand what is happening in their communication with doctors, and having a clear understanding of their diagnosis, prognosis and treatment [53].

Previous research also suggests that while HPs communicating in an additional language are generally proficient in English [54], they may experience difficulties with some aspects of their L2 such as pronunciation, accent, pace and intonation [55, 56]. However, bilingual doctors were reported to try to use strategies, such as learning how to pronounce words in a North American manner, repeating their sentences, or changing the pace of their speech, to overcome these barriers and accommodate to their patients [46]. Although the present data do not establish that these factors caused difficulties in understanding, they reinforce the importance of checking understanding of messages that contain expressions of uncertainty and risk, not only with bilingual patients but also—when working in an Australian, English‐speaking healthcare system—with native English speakers from culturally diverse backgrounds (e.g., Singapore, South Africa). HPs should also pay attention to expressions of uncertainty used by their patients, because these may reflect an unspoken request for reassurance, especially for older patients. For CALD patients, their use of epistemic adverbs may reflect insufficient knowledge of English or uncertainty regarding cultural norms and expectations in healthcare.

Reassuringly, the HPs used CAT strategies effectively to adapt to their patients' conversational needs, and they mostly demonstrated positive accommodative behaviours. Although we targeted only those parts of the talk that involved (un)certainty regarding the patient's health or diagnosis, on multiple occasions HPs consistently used combinations of strategies (see Table 10). Their use of multiple strategies created communication patterns, such as ‘small talk‐providing diagnosis’ sandwiches. Results of our post‐interview survey showed that the patients rated the quality of the interactions they had with their HP highly (mean = 4.9; 5 = Definitely), confirming that they were happy. The results of our study are consistent with findings from a quantitative study of CAT strategies preferred by patients across four countries, which found that Australian patients prefer to be actively engaged in dialogue with their health professionals and have their emotions acknowledged by them [53].

### 5.1 Limitations and Future Research

We recognise that the recorded consultations were snapshots of a communicative interaction between a patient and an HP at Time 1. It is conceivable that misunderstandings emerged following the consultations. However, because no follow‐up was possible, it remains unknown how the patient's understanding informed their future actions, such as subsequent consultations with their own GP about the same health concern (the uncaptured Time 2). Future research incorporating longitudinal follow‐up could examine how uncertainty is negotiated beyond the initial ED encounter and how its communication shapes later health behaviours and outcomes.

We further acknowledge the heterogeneous nature of our sample. The recording of the consultations was entirely dependent on the patients who presented (and consented) and the HPs attending on the day. Consequently, the HP sample was varied and consisted of doctors, nurses and physiotherapists, all of whom follow different professional protocols and communication practices when interacting with patients in the ED. These differences may have indirectly influenced their use of epistemic adverbs. In turn, patients presented with a wide range of medical conditions and varying levels of health literacy, which also may have affected their use of epistemic adverbs. In addition, levels of English proficiency varied among bilingual patients. While this heterogeneity introduced variability, it also reflects the naturalistic design of the study and the real‐world nature of ED communication, where language barriers, clinical uncertainty and time pressures are negotiated in real time. Future research could address these limitations by focusing on a specific group of HPs (e.g., nurses or doctors) or comparisons between HP groups, to examine how they may use accommodation strategies differently when communicating uncertainty.

As previously stated, we excluded two consultations from the analysis because they were brokered: the patient spoke little or no English. One further consultation was excluded not only because it was not health focused, but because part of the interaction was conducted with the assistance of an interpreter. Brokered and interpreter‐assisted consultations where there is medical uncertainty are important to understand better with the aim to mitigate against misunderstanding and improve patient outcomes. Future research may wish to focus on the management of uncertainty in brokered versus interpreter‐supported consultations.

Future research should further examine the most common epistemic adverbs and other epistemic expressions used in health communication. Our findings suggest that uncertainty adverbs may be a good starting point for further work, as patients used them more often than confident adverbs. This greater use of uncertainty adverbs may reflect patients' need for reassurance, dovetailing with the use of reassurance strategies by HPs [57, 58]. It may also indicate attempts by patients to communicate their uncertainty regarding symptoms, diagnoses or treatment outcomes. This interpretation is consistent with Mishel's Uncertainty in Illness Theory, which proposes that uncertainty arises when individuals experience difficulty interpreting illness‐related events because of ambiguity, complexity, unpredictability or insufficient information [59, 60]. It also aligns with the Theory of Motivated Information Management, which conceptualises uncertainty as a catalyst for information‐management and support‐seeking behaviours [61]

Additionally, a larger quantitative study on the use of uncertainty adverbs in language‐discordant communication could help HPs better understand bilingual patients' usage patterns, enhancing communication and promoting higher patient satisfaction with communication and better patient outcomes. Such a study may also offer a further expansion of Stage 6 in CAT research, which focuses on processes and mechanisms that mediate the effects of accommodation on conversational and social outcomes [30, 62]. Results of a study on perceptions of police‐civilian encounters conducted in the UAE and the United States showed that when the public perceived police officers as accommodating, they were more inclined to comply with the officers' directives and more willing to report crime. Perceived accommodation led to increased trust in police [63]. In a similar vein, future research on uncertainty in health communication may examine whether perceived accommodation from HPs increases patients' willingness to accept medical uncertainty and whether it also results in increased trust in HPs. If patients' perceptions can be positively adjusted, this could have beneficial effects for bilingual patients and other minority groups, not least of which First Nations Australians.

It is important to note that it is unknown whether, in the recorded consultations, patients fully discussed their health concerns. HPs need to be aware that this may be the case and check with their patients. Future research may wish to consider including a follow‐up session with patients and HPs (e.g., in the form of semi‐structured interviews) to check the patient's understanding of the uncertainty manifested in consultation and whether HPs and patients agree on how uncertainty was understood by both parties. A design similar to the one used by Chevalier et al. [26] in their study on patients' and pharmacists' perspectives on communication strategies used by hospital pharmacists would be suitable. Alternatively, participants can be asked to make judgements of vignettes based on real‐life consultations, to add to our understanding of the judgements people make about the communication of uncertainty in health interactions.

## 6 Conclusion

Both HPs and patients used epistemic adverbs to communicate uncertainty and risk. Overall, in the analysed episodes, HPs used different strategies to accommodate their patients in the parts of the consultations where uncertainty was manifested by epistemic adverbs. However, there were instances when it was unclear whether epistemic adverbs used by HPs were understood by their patients as intended. This is concerning, because the misunderstandings that can result may put patients at risk and contribute to poorer patient outcomes. To ensure clarity of their messages to patients, HPs should reflect on their use of epistemic adverbs, and how these are variably understood depending on their patient's cultural and linguistic background.

### 6.1 Practical Implications

The implications of our findings for HPs are threefold. First, HPs should be especially mindful when using risk and uncertainty expressions in their consultations. For HPs who practice in their other language (i.e., not their L1), such mindful practice is vital given that their own use and understanding of uncertainty expressions may differ from that of their patients. Second, when communicating with patients, especially when it is evident that the patient speaks another language as their L1 and when the health information that is conveyed to them must be clearly understood by the patient (e.g., treatment options, medication instructions), HPs are advised to carefully check their patients' understanding, repeat important messages and/or avoid using epistemic expressions where possible. Finally, the recommendations above should not be confined to linguistically or culturally discordant healthcare contexts. When we speak the same language, perhaps especially when we are native or fluent speakers, we tend to assume that the words we use are understood, as intended, by others. They may not be, and it is important for HPs to be mindful of nuances in meaning, especially when communicating uncertainty, even when both HP and patient share the same L1.

## Author Contributions

**Vanda Nissen:** conceptualisation, methodology, funding acquisition, writing – original draft, writing – review and editing, formal analysis, investigation. **Renata F. I. Meuter:** supervision, writing – review and editing, conceptualisation, methodology, funding acquisition. **Michelle Riedlinger:** supervision, writing – review and editing.

## Ethics Statement

Ethics approval was obtained from the Metro South Human Research Ethics Committee and the Human Research Ethics Committee at Queensland University of Technology (Ethics approval number HREC/2021/QMS/73181).

## Conflicts of Interest

The authors declare that the research was conducted in the absence of any commercial or financial relationships that could be construed as a potential conflict of interest.

## Data Availability

Due to the nature of this research, participants of this study did not agree for their data to be shared publicly.

## Footnotes

^1^
Language discordant consultations are those in which either the patient or the provider, or both, communicate in a language that is not their dominant, preferred, or first language (L1). When both parties communicate in their preferred, typically first and dominant language, there is language concordance.

^2^
We should note that different epistemic expressions such as modal verbs, epistemic parentheticals and hedging, were used across all 53 conversations.

^3^
Companions' and chaperones' speech was excluded from the analysis.

^4^
We acknowledge that ‘Chinese’ may refer to several spoken varieties. We have retained this term because it reflects Australian NAATI conventions, where ‘Chinese’ is used for written translation certification, while spoken interpreting credentials specify the relevant variety, such as Mandarin or Cantonese.

## 1

See Table A1, A2, A3, A4, A5.

**Table A1:** Overview of the patient consultations (*n* = 39) according to the interlocutors (patients (P) and health practitioners (HPs), their language background (bilingual (Bil) or monolingual (Mono), language spoken during the consultation (L1 or L2), the Resulting language context (discordant or concordant) and, for each language context, the mean length of consultations, and the mean number of words, per mean length of conversations.

	Interlocutor		Language context		Mean length of consult	Mean #words/mean length of consult
	Patient (P) (*N* = 34)	HP (*N* = 28)	Discordant	Concordant		Patient (P) (*N* = 34)
Language background	Bil (L2)	Bil (L2)	*n* = 3		6.42	123.60
		Mono (L1)	*n* = 10		8.55	126.60
	Mono (L1)	Bil (L2)	*n* = 12		8.56	106.33
		Mono (L1)		*n* = 14	10.65	124.50

*Note:* 1: All consultations took place in English; however, for bilingual patients and bilingual HPs, English was their L2. Language (dis)cordance is defined in relation to whether the language spoken by the interlocutor is their L2. That is, if a bilingual patient (English L2) consults with a monolingual HP (English L1), the consultation is language discordant, as is a consultation between a monolingual patient (English L1) and a bilingual HP (English L2). 2: There were few consultations (*N* = 3) in which both patient and HP spoke their L2 (English).

**Table A2:** Demographic details for patients.

	Patient	Sex	Age	Language background
1.	Patient 1	Male	70	Monolingual
2.	Patient 2	Male	43	Monolingual
3.	Patient 3	Female	34	Bilingual
4.	Patient 4	Female	59	Bilingual
5.	Patient 5	Male	24	Monolingual
6.	Patient 6	Male	75	Monolingual
7.	Patient 7	Male	76	Monolingual
8.	Patient 8	Female	20	Bilingual
9.	Patient 9	Female	47	Bilingual
10.	Patient 10	Female	32	Monolingual
11.	Patient 11	Male	73	Monolingual
12.	Patient 12	Female	51	Monolingual
13.	Patient 13	Female	53	Monolingual
14.	Patient 14	Male	52	Bilingual
15.	Patient 15	Female	71	Bilingual
16.	Patient 16	Male	23	Monolingual
17.	Patient 17	Male	25	Monolingual
18.	Patient 18	Female	37	Bilingual
19.	Patient 19	Male	44	Monolingual
20.	Patient 20	Male	72	Bilingual
21.	Patient 21	Male	68	Bilingual
22.	Patient 22	Male	77	Bilingual
23.	Patient 24	Male	45	Monolingual
24.	Patient 25	Male	76	Monolingual
25.	Patient 26	Female	22	Bilingual
26.	Patient 27	Male	37	Monolingual
27.	Patient 28	Male	62	Monolingual
28.	Patient 29	Female	56	Monolingual
29.	Patient 30	Male	70	Monolingual
30.	Patient 31	Male	57	Monolingual
31.	Patient 32	Female	42	Monolingual
32.	Patient 33	Male	28	Monolingual
33.	Patient 34	Female	80	Monolingual
34.	Patient 35	Female	61	Monolingual

**Table A3:** Patients' language background.

		Patient		
		F	M	
Lang. Backgrd	Bilingual	7	4	11
		[20, 71]	[52, 77]	
	Monolingual	7	16	23
		[32, 80]	[24, 76]	
Total		14	20	34

*Note:* The age range is given in brackets.

**Table A4:** Demographic details for HPs.

	Occupation	Sex	Age	Language background
1.	Doctor	Male	23	Bilingual
2.	Nurse	Male	46	Bilingual
3.	Doctor	Female	49	Monolingual
4.	Doctor	Male	26	Monolingual
5.	Doctor	Male	33	Bilingual
6.	Doctor	Female	25	Bilingual
7.	Doctor	Female	36	Bilingual
8.	Doctor	Male	25	Bilingual
9.	Doctor	Male	32	Monolingual
10.	Doctor	Male	26	Bilingual
11.	Doctor	Male	29	Monolingual
12.	Doctor	Female	34	Monolingual
13.	Nurse	Female	41	Monolingual
14.	Doctor	Male	25	Monolingual
15.	Doctor	Female	26	Monolingual
16.	Nurse	Female	62	Monolingual
17.	Doctor	Male	56	Monolingual
18.	Nurse	Female	33	Monolingual
19.	Doctor	Female	30	Monolingual
20.	Doctor	Male	29	Bilingual
21.	Doctor	Male	24	Bilingual
22.	Physiotherapist	Male	39	Monolingual
23.	Nurse	Female	28	Monolingual
24.	Nurse	Male	22	Bilingual
25.	Doctor	Female	44	Monolingual
26.	Doctor	Female	28	Monolingual
27.	Nurse	Female	60	Monolingual
28.	Physiotherapist	Female	25	Monolingual

**Table A5:** HPs' language background and age range.

		Occupation						
		Doctor		Nurse		Physio		Total
		F	M	F	M	F	M	
Lang. Backgrd	Bilingual	2	6	—	2	—	—	10
		[25, 36]	[23, 33]					
	Monolingual	6	5	5	—	1	1	18
		[26, 49]	[25, 56]	[28, 62]		[25]	[39]	
Total		8	11	5	—	1	1	28

*Note:* The age range is given in brackets.
